# Integration and comparison of different genomic data for outcome prediction in cancer

**DOI:** 10.1186/s13040-015-0065-1

**Published:** 2015-10-29

**Authors:** Hugo Gómez-Rueda, Emmanuel Martínez-Ledesma, Antonio Martínez-Torteya, Rebeca Palacios-Corona, Victor Trevino

**Affiliations:** 1 Departamento de Investigación e Innovación, Grupo de Investigacion en Bioinformatica, Escuela de Medicina, Tecnológico de Monterrey, Monterrey, Nuevo León 64849 Mexico; 2 Centro de Investigación Biomédica del Noreste, Instituto Mexicano del Seguro Social, Monterrey, Nuevo León 64720 Mexico

**Keywords:** Survival, Cancer, Genomics, TCGA

## Abstract

**Background:**

In cancer, large-scale technologies such as next-generation sequencing and microarrays have produced a wide number of genomic features such as DNA copy number alterations (CNA), mRNA expression (EXPR), microRNA expression (MIRNA), and DNA somatic mutations (MUT), among others. Several analyses of a specific type of these genomic data have generated many prognostic biomarkers in cancer. However, it is uncertain which of these data is more powerful and whether the best data-type is cancer-type dependent.

Therefore, our purpose is to characterize the prognostic power of models obtained from different genomic data types, cancer types, and algorithms. For this, we compared the prognostic power using the concordance and prognostic index of models obtained from EXPR, MIRNA, CNA, MUT data and their integration for ovarian serous cystadenocarcinoma (OV), multiform glioblastoma (GBM), lung adenocarcinoma (LUAD), and breast cancer (BRCA) datasets from The Cancer Genome Atlas repository. We used three different algorithms for prognostic model selection based on constrained particle swarm optimization (CPSO), network feature selection (NFS), and least absolute shrinkage and selection operator (LASSO).

**Results:**

The integration of the four genomic data produced models having slightly higher performance than any single genomic data. From the genomic data types, we observed better prediction using EXPR closely followed by MIRNA and CNA depending on the cancer type and method. We observed higher concordance index in BRCA, followed by LUAD, OV, and GBM. We observed very similar results between LASSO and CPSO but smaller values in NFS. Importantly, we observed that model predictions highly concur between algorithms but are highly discordant between data types, which seems to be dependent on the censoring rate of the dataset.

**Conclusions:**

Gene expression (mRNA) generated higher performances, which is marginally improved when other type of genomic data is considered. The level of concordance in prognosis generated from different genomic data types seems to be dependent on censoring rate.

**Electronic supplementary material:**

The online version of this article (doi:10.1186/s13040-015-0065-1) contains supplementary material, which is available to authorized users.

## Background

Cancer is a public health problem worldwide due to its high prevalence and mortality rates [1]. In the year 2012 alone, there were 14.1 million new cases of cancer, from which 8.2 million resulted in death [2]. Moreover, projections estimate a 20 % and 40 % increase of cancer cases for the years 2020 and 2030, respectively relative to 2010. The cancers of breast and lung cancers are expected to remain within the top cancer diagnoses and leading causes of cancer-related death [3].

Patient prognosis has a fundamental role in treatment, and research [3–8]. As a result, many prognostic biomarkers have been proposed using a wide range of biological features, such as genomic [9], proteomic [10], metabolomic [11], pathological [12], imaging [13], and psychological features [14]. From these, genomic features are currently the most used in biomarker discovery analyses [15], mainly due to significant efforts made by the National Cancer Institute and the National Human Genome Research Institute, which resulted in The Cancer Genome Atlas (TCGA) project [16]. TCGA has gathered information from several sources of genomic data on over 30 cancer types [17]. Large-scale technologies, like next-generation sequencing and microarrays, have been used to obtain DNA copy number alterations (CNA), mRNA expression (EXPR), microRNA expression (MIRNA), DNA methylations, and DNA somatic mutations (MUT), among others. These data have already been used to propose many cancer prognostic signatures [17–24].

Identifying which source of genomic data, or combination, generates the most powerful prognostic biomarker could help to describe cancer etiology [16, 19, 20]. However, some studies have generated inconsistent results across cancers when evaluating distinct sources of genomic data for prognosis [19, 20], probably because of the use of different algorithms. Thus, it is not clear which type of data is the best at predicting cancer prognosis or whether combinations of data types provide some improvement. For example, it has been shown that no significant improvement is obtained adding any genomic measurement once EXPR data and clinical covariates were included in the model [19] using principal components, partial least squares, and a penalization algorithm. On the other hand, a similar study showed that all clinical outcomes were better predicted when integrating multi-layers of genomic data [20] using a graph-based algorithm while others suggest that the clinical improvement of genomic data is limited in magnitude and on cancer types [21] using diverse classification algorithms.

Given the lack of concordance on methods and genomic data provides the best prognostic results and its utility, our purpose is to characterize the prognostic power of models obtained from different genomic data types, cancer types, and algorithms. For this, we tested the prognostic and concordance index of models obtained by three different algorithms from EXPR, MIRNA, CNA, MUT data and their integration for ovarian serous cystadenocarcinoma (OV), multiform glioblastoma (GBM), lung adenocarcinoma (LUAD), and breast cancer (BRCA) datasets from the TCGA repository. The algorithms used are based on very different properties to search for diverse solutions attempting to derive conclusions at certain independency of the algorithms. We used constrained particle swarm optimization (CPSO) [22], which explores combinations of features irrespectively of its biological connections, network feature selection (NFS) [23] that explores combination of features integrating protein-protein interaction information, and the least absolute shrinkage and selection operator (LASSO) [24] that explores penalized models.

## Methods

**Fig. 1:**
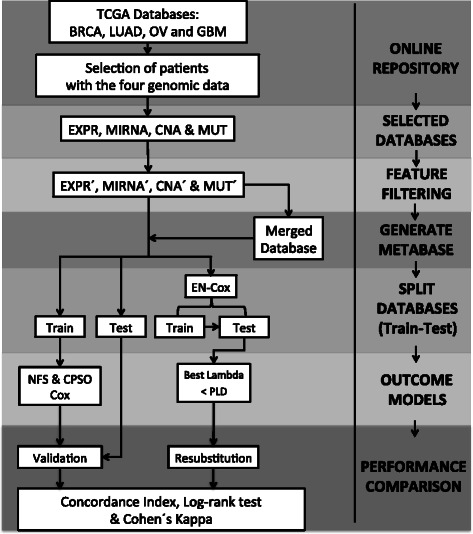
Overview of the methodology. TCGA: The Cancer Genome Atlas. BRCA: Breast Cancer. LUAD: Lung Adenocarcinoma. OV: Ovary Cystadenocarcinoma. GMB: Glioblastoma Multiform. EXPR: Gene Expression. MIRNA: micro RNAs. CNA: Copy Number Alteration. MUT: Somatic Mutations’. Stands for Filtered Data. EN: Elastic-Net LASSO (Least Absolute Shrinkage and Selection Operator). NFS: Network Feature Selector. CPSO: Constrained Particle Swarm Optimization. c-index: Concordance Index

The methodology is summarized in Fig. 1. Briefly, samples from the four cancer types that fulfill a specific inclusion criterion were selected. Features from each database and source were filtered. The resulting four databases were then merged into a metabase (MERGE) for comparisons with single-sourced databases. Predictive models were obtained for each database using three feature selection algorithms that generate a unique model. Finally, the performance of the models was evaluated using the concordance index (c-index) [25].

### Database selection

The data used in this study was downloaded in April 2013 from the TCGA data portal (https://tcga-data.nci.nih.gov/tcga/) including level 2 (MUT) and level 3 (EXPR, MIRNA, and CNA) data. CNA was segmented by regions per sample using the GISTIC algorithm [26]. EXPR and MIRNA data were quantile-normalized before use. Using the TCGA-ID, a tag unique to each subject, only those subjects with available EXPR, MIRNA, CNA, and MUT data were used. The results published here are in whole or part based upon data generated by The Cancer Genome Atlas pilot project established by the NCI and NHGRI. Information about TCGA and the investigators and institutions who constitute the TCGA research network can be found at http://cancergenome.nih.gov/.

### Feature filtering

We filtered features to reduce complexity, to avoid the use of invariant information, and to balance the number of features from each source avoiding to remove predictive information. MIRNA and EXPR databases were filtered using a correlation and quantization strategy. First, features without absolute Spearman correlation coefficient larger than 0.6 were excluded. Second, to remove invariant genes, we split the data into five uniform segments and only those genes having counts in more than two segments were used. For CNA data, we used the 10 % probes having the most significant *p*-values using the univariate log-rank test from a Cox proportional hazard model splitting the linear predictor at the median. For MUT data, we used the 11.4 % of OV, 12.2 % of LUAD, 9.9 % of BRCA and 30.4 % of GBM, of the most frequently mutated genes. Using LUAD as an overall validation of the filtering procedure, we observed that using LASSO and all features in CNA, EXPR, MIRNA and MERGE, the results were 61, 77, 76, and 78 of concordance index respectively, which are very close to those observed after the filtering.

### Metabase generation

A fifth dataset (MERGE) was constructed per cancer type by merging their corresponding EXPR, MIRNA, CNA, and MUT filtered databases. This allowed a direct comparison on which data source is best selected in the presence of other sources. Furthermore, the metabases permitted the identification of predictive models with features from different sources, and compare such compound models with single-source models.

### Feature selection algorithms

We used a multivariate Cox proportional hazard model for the three feature selection algorithms. Beta coefficients were calculated by optimizing either the log-likelihood (NFS and CPSO) or a penalized maximum likelihood function (LASSO) through several iterations using bootstrap (NFS and CPSO) or a 10-fold cross-validation (LASSO) scheme [27, 28]. In the case of NFS and CPSO, only two- thirds of the population was used for training while the remaining was used to perform a blind test. Bootstrap consists in randomly sampling the population using a similar fraction per strata in the resampled sets [29]. For MUT databases, we relied on resubstitution because mutation data is sparse where only small number mutations are observed per gene, which may generate sets of training samples with no mutations at all.

### Constrained particle swarm optimization (CPSO)

Particle swarm optimization algorithms are based on the biological behavior of swarms. Concisely, these algorithms create a swarm of particles with random positions and velocities. The positions represent parameters of the problem to solve. The particles will update their velocity and position depending on their performance, iteratively. The performance is a function that evaluates the particle position relatively to the swarm [22]. We have customized PSO (CPSO) to handle feature selection problems from large genomic datasets [30]. This algorithm uses a user-defined number of features, *k*, to generate efficiently a subset of features that is used as the survival model. We used *k* = 5 and 500 iterations. We ran the algorithm 1,000 times. Models generated contained between 8 and 10 genes. We used the model with the highest c-index estimated by bootstrapping.

### Network feature selection (NFS)

Network Feature Selection (NFS) is based on the exploration of protein-protein interaction networks to select features resulting in more biologically coherent models [23]. NFS has recently been used to generate multi-cancer biomarkers [23]. Briefly, each feature is evaluated individually by the p-value of an univariate Cox proportional hazards model. Each gene is then considered as a survival model. Each model grows by considering all possible neighbors according to the interactions provided by a network. The top 5 % of these grown models having higher performance are selected to grow in the next iteration. This procedure is carried on until no model can be further grown, or until 10 iterations. The protein-protein interaction network used was downloaded from the human protein reference database (HPRD, http://www.hprd.org/). Genes having more than 1,000 connections are not allowed to grow (for example, the UBC gene). For MIRNA data, the interactions between miRNA and mRNA were considered as surrogate interactions for the network, where the mRNA was replaced by the miRNA that regulates it. In order to identify the targets of each miRNA and create the miRNA/protein-protein interaction network, the miRTarDatabase (http://mirtarbase.mbc.nctu.edu.tw/) was used. In the MERGE dataset, the gene/protein connections were used irrespective of the data type.

### Least absolute shrinkage and selection operator (LASSO)

LASSO is a well-known widely used feature selection algorithm, particularly when the number of samples is considerably smaller than the number of features. This algorithm performs a coefficient penalization in which only well-associated features emerge [28]. The best model containing around 10 features was used.

### Performance evaluation

Models were evaluated and compared using the concordance index (c-index) and the p-value of the log-rank. The c-index was used to assess the prediction power of the survival model [25, 31]. The log-rank test was used to determine whether low- and high-risk groups were significantly different from each other [25, 31]. These statistics were estimated using the blind test subset for the models generated with CPSO and NFS, or using re-substitution for the models generated with LASSO. To compare the agreement of prognostic prediction of two models, we used the Cohen's kappa statistic in R implemented within the package *fmsb* [32]. For this, we split the prognostic index by the median. The prognostic index is the linear predictor of the exponential function in the Cox model [27].

## Results

We used OV, LUAD, BRCA, and GBM datasets that had at least 100 subjects with EXPR, MIRNA, CNA, and MUT data in the TCGA repository at the time of accession. A brief description of the technologies and clinical and demographic information is included in Additional file 1: Table S1 and Additional file 2: Table S2. The number of features of each dataset before and after filtering is detailed in Table 1.

**Table 1:** Number of features used by the feature selection algorithms

	Before filtering				After filtering			
	OV	LUAD	BRCA	GBM	OV	LUAD	BRCA	GBM
EXPR	12,042	20,502	17,787	12,042	1,203	4,632	3,836	1,204
MIRNA	705	1,046	1,046	534	108	578	587	534^a^
CNA	24,174	24,174	23,862	24,117	2,417	2,417	2,417	2,417
MUT	12,042	20,502	11,929	20,502	1,371	2,500	1,175	6,241

^a^Not filtered because of low number of remained filtered features

**Fig. 2:**
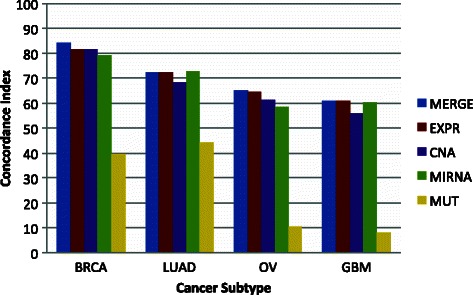
Performance of the models generated with different genomic data sorted by the cancer subtypes. BRCA: Breast Cancer. LUAD: Lung Adenocarcinoma. OV: Ovary Cystadenocarcinoma. GMB: Glioblastoma Multiform. EXPR: Gene Expression. MIRNA: micro RNAs. CNA: Copy Number Alteration. MUT: Somatic mutations

**Fig. 3:**
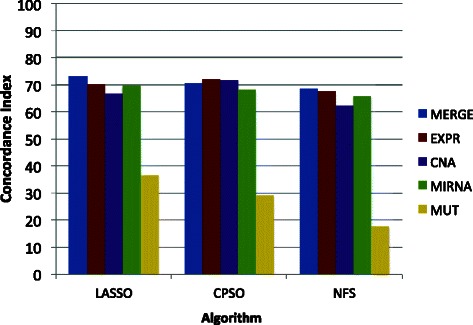
Performance of the models generated with different genomic data sorted by the used algorithms. EXPR: Gene Expression. MIRNA: micro RNAs. CNA: Copy Number Alteration. MUT: Somatic mutations. LASSO (Least Absolute Shrinkage and Selection Operator). NFS: Network Feature Selector. CPSO: Constrained Particle Swarm Optimization

The results of the c-index and the log-rank test of all cancer types, data types, and algorithms are shown in Table 2. From the genomic data types, we observed better prediction in EXPR closely followed by MIRNA and CNA depending on the cancer type and method (Figs. 2 and 3). In our tests, mutation data generated poor predictions. In average, the results of the MERGE dataset were marginally more predictive that any of the other data types (Figs. 2 and 3). Within the MERGE dataset, we explored which of the dataset was more important. The Table 3 shows the number of features per data type used by the best model in the MERGE database. The results further support that EXPR is the preferred data (54 % of the features) when all other data is present. Surprisingly, EXPR was followed by CNA (27 %) and then MUT (14 %) while MIRNA data was almost not used (6 %).

**Table 2:** Concordance index and log-rank test of all models

Cancer type	Algorithm	EXPR	MIRNA	CNA	MUT	MERGE
OV	CPSO	66^b^	61^b^	64^c^	10^c^	65^c^
	NFS	60^a^	53	56^b^	11^c^	63^c^
	LASSO	68^c^	62^c^	64^c^	-	68^c^
	Average	65	59	61	10	65
LUAD	CPSO	74^b^	70	74^b^	52^c^	75^b^
	NFS	71^b^	73^b^	65^a^	29^b^	64
	LASSO	72^c^	75^c^	66^c^	52^c^	78^c^
	Average	72	72	68	44	72
BRCA	CPSO	85^c^	82^c^	92	38^c^	83^c^
	NFS	79	76	70	28^c^	84
	LASSO	81^c^	80^b^	83^c^	53^c^	86^c^
	Average	82	80	82	40	84
GBM	CPSO	63^c^	59^c^	57^b^	16^c^	59
	NFS	60^c^	61^c^	58^b^	3^b^	63^c^
	LASSO	60^c^	61^c^	53^c^	5	61^c^
	Average	61	61	56	8	61
Overall	CPSO	72	68	72	29	71
	NFS	67	66	62	18	69
	LASSO	70	70	66	37	73
	Average	70	68	67	27	71

^a,b,c^Indicate models whose Kaplan-Meier curves were statistically different at 0.05, 0.01, and 0.001 level respectively using the log-rank test. For this, the population was split by the median using the prognostic index (linear predictor of the Cox model). “-” indicates that no models were generated

**Table 3:** Feature source distribution for MERGE models

Algorithm	Dataset	Size	EXPR	MIRNA	CNA	MUT
CPSO	BRCA	10	6	0	3	1
	LUAD	9	6	0	3	0
	GBM	10	2	2	1	5
	OV	10	6	0	4	0
	Total	39	51 %	5 %	28 %	15 %
NFS	BRCA	4	0	0	4	0
	LUAD	4	3	0	1	0
	GBM	9	4	0	5	0
	OV	9	4	0	4	1
	Total	26	42 %	0 %	54 %	4 %
LASSO	BRCA	11	4	0	2	5
	LUAD	9	3	3	1	2
	GBM	13	10	1	1	1
	OV	10	10	0	0	0
	Total	43	63 %	9 %	9 %	19 %
Overall		216	54 %	6 %	27 %	14 %

Percentages were rounded to closest integer

We observed higher predictions in BRCA, followed by LUAD, OV, and GBM having an average c-index around 0.82, 0.71, 0.63, and 0.60, respectively. These comparisons agree with recent results of multi-cancer gene expression biomarkers [23]. In BRCA and OV, CNA data were more predictive than MIRNA. In LUAD and GBM, the c-index of MIRNA was higher than CNA and comparable with EXPR data. Although the results of MUT were poor, in BRCA and LUAD the predictions were higher than in OV and GBM even though we used more genes in those cancer types.

We observed similar c-index values between LASSO and CPSO but smaller c-index values in NFS (Fig. 3). The MERGE data was more predictive in LASSO and NFS but not in CPSO where EXPR was the best. CNA was clearly more predictive in CPSO than in LASSO and NFS (Fig. 3).

**Fig. 4:**
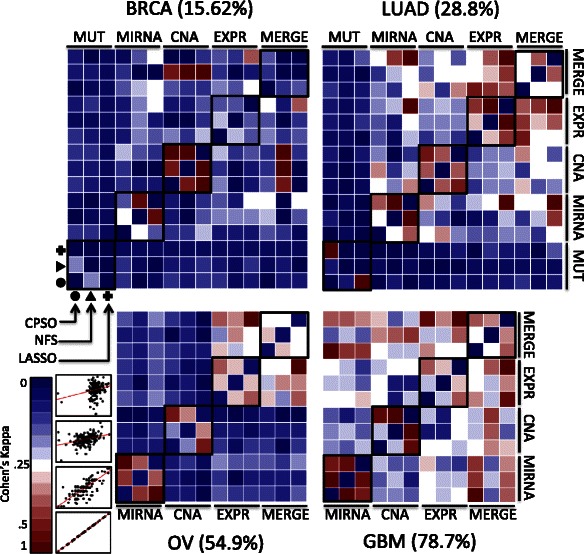
Agreement in the prognostic prediction by cancer type and data type. The figure shows the Cohen’s Kappa agreement of the risk assessment based on the median of the prognostic index generated by each model. Each heatmap shows the comparison of the models generated between data types and algorithms. Cells shown in squares correspond to the comparisons between the models of the three algorithms (CPSO, NFS, LASSO) for the same data type. The event proportion in each cancer type is shown in parenthesis. Within heatmaps, blue colors denote lower *kappa* value, white denotes intermediate values, and red denotes high kappa values. For comparison, the scatter-plots shown aside the color-coding corresponds to examples of prognostic indexes pairs having 0, 0.25, 0.5, and 1 of kappa values. MUT data did not generate risk groups in OV and GBM and were omitted. EXPR: Gene Expression. MIRNA: micro RNAs. CNA: Copy Number Alteration. MUT: Somatic mutations. LASSO (Least Absolute Shrinkage and Selection Operator). NFS: Network Feature Selector. CPSO: Constrained Particle Swarm Optimization. BRCA: Breast Cancer. LUAD: Lung Adenocarcinoma. OV: Ovary Cystadenocarcinoma. GMB: Glioblastoma Multiform

We also compared whether the predictions made by models concur. We used the Kappa statistic that measures the level of concordance of two predictors. Values of Kappa close to 0 correspond to random agreements whereas values close to 1 represent perfect agreement. The results show that MIRNA, CNA, and EXPR models have acceptable agreement in LUAD, OV, and GBM irrespective of the method (Fig. 4). In BRCA, we found agreement in CNA models and partially in MIRNA. In addition, MIRNA slightly agrees with CNA in LUAD and with EXPR in GBM. In general, however, the predictions made by different types of data disagree.

## Discussion

Our objective was to compare and characterize the prognostic level of different genomic data sources in cancer. For this, we analyzed four important cancer types (BRCA, LUAD, OV, GBM) that have diverse survival times. The analysis was performed using a feature selection method trained with a specific data type. For feature selection, we used three methods (LASSO, NFS, CPSO). For the data types, we used the genomic data available at the time of the analysis (EXPR, MIRNA, CNA, MUT) and the union of these (MERGE).

Overall, MERGE data was the most predictive across the four cancer types (Fig. 2) and the three algorithms (Fig. 3). This result is sensible because MERGE contained all other data types. Nevertheless, in some cases MERGE was not the best. This was the case in CPSO whose performance could be influenced by the increased number of features.

From the genomic data types (EXPR, MIRNA, CNA, MUT), the best performance was obtained with EXPR (Figs. 2 and 3). The gene expression is the result of complex dynamic interactions between all components of the system (genome, proteome, metabolome, and environment). Consequently, any type of alterations or stimuli is likely to influence EXPR. CNA and MIRNA followed EXPR in performance (Figs. 2 and 3 and Table 2). CNA represents changes in DNA, which are presumably less dynamic than EXPR. Nevertheless, the CNA performance was surprisingly comparable to EXPR suggesting that a considerable component of the survival is dictated by CNA. The performance of MUT data was poor when compared to EXPR, MIRNA, and CNA. Some issues are known relative to this lack of prediction. First, mutation frequencies per gene are generally low suggesting that mutation data is highly disperse [21]. Second, the combination of sparseness and binary data (mutated or not mutated) may generate difficulties in the Cox model fitting. Third, the reports of mutation frequencies do not commonly find associations with survival [33–36].

We did not observe big differences in performance relative to the algorithm used. CPSO seems to show consistent and highly competitive results, but LASSO seems to report slightly higher results while NFS seems to produce lower performance.

The prognostic values provided by different methods for the same data type were remarkably high suggesting that the algorithm used is a minor source of differences (Fig. 4). However, the observation of the lack of similitude in risk prediction between different data types was surprising and an important result of our study (Fig. 4). It is known that the precision of the prognostic values is highly influenced by the proportion of censoring [37]. We observed higher similitudes between the prognostic values generated by different data types in GBM where the proportion of censoring is the lowest (21.3 %) and lower similitudes in BRCA where the proportion of censoring is the highest (84.4 %). We also observed that the prediction in BRCA is high (around 0.8 of c-index) while in GBM is low (around 0.6 of c-index). The c-index measures how well the model fit the censoring data while kappa measures the consistency of two predictions. We showed that these properties seem to be highly influenced by the proportion of events. More research is needed to determine the lack of consistency.

We used four cancer types, three algorithms, and five data types. There may be some level of interaction between these three components. For instance, the performance of MIRNA data was higher in LUAD and the performance of NFS was generally lower using CNA data. We did not study thoroughly the possible parameters combinations within each algorithm, nor many potential schemes of data type filtering and processing. However, our results suggest some tendencies and the results should be similar to other cancer types and algorithms in similar circumstances to those tested here.

## Conclusions

The integration of genomic data produced survival models were marginally higher in performance than those from single genomic data, specially those of mRNA. From the genomic data, the mRNA gene expression generated the highest predictive models and were preferred in models that integrate the four types of genomic data. CNA and miRNA data followed mRNA in performance while mutation data poorly predicted survival. The risk prediction of survival models of different types of data disagrees and the level of agreement seems to be related to the censoring rate.

## Additional files

Additional file 1: Table S1. Technology used per cancer and genomic data type. (XLSX 8 kb) Additional file 2: Table S2. Clinical data per cancer type. (XLSX 9 kb)

## Abbreviations

CNA: Copy number alteration
EXPR: Gene expression of mRNA
MIRNA: Expression of miRNA
MUT: Somatic mutations
MERGE: Database that contains the four genomic data of the patients
OV: Ovarian serous cystadenocarcinoma
GBM: Multiform glioblastoma
LUAD: Lung adenocarcinoma
BRCA: Breast cancer
TCGA: The Cancer Genome Atlas
CPSO: Constrained particle swarm optimization
NFS: Network feature selector
LASSO: Least absolute shrinkage and selection operator
c-index: Concordance index
